# Ovarian Tumors

**Published:** 1862-03

**Authors:** Wm. H. Byford

**Affiliations:** Professor of Obstetrics and Diseases of Women and Children, in the Medical Department, Lind University, Chicago, Illinois


### CHICAGO MEDICAL EXAMINER

N. S. DAVIS, M. D., Editor.---FRANK W. REILLY, M. D., Ass't Editor.

VOL. IV.] MARCH, 1862. [NO. III

### Original Contributions.

ARTICLE VIII.

### OVARIAN TUMORS.

By WM. H. BYFORD, A. M., M. D.,

PROFESSOR OF OBSTETRICS AND DISEASES OF WOMEN AND CHILDREN, IN THE MEDICAL DEPARTMENT, LIND UNIVERSITY, CHICAGO, ILLINOIS.

### V.—Treatment of Ovarian Tumors.

It is not necessary to interfere, in any manner, with some cases of ovarian dropsy. Indeed, I think it would be nearly, if not always right to let all cases alone that did not threaten the life of the patient with material curtailment. There are many instances which advance slowly, or remain stationary for a great many years, and prove but an inconvenience. We would not be justified in active interference in these cases; much less should we do anything directly, for cases in which independent complications of a fatal character exist; *e. g.*, phthisis or cancer. When, however, the disease is making obvious progress, and particularly when the advance is sufficiently rapid to leave but little doubt of its proving fatal within the average time of their duration, we are bound to make every effort within our power to save or prolong, as much as possible, the life of our patient.

When treatment becomes necessary, and we cast about for remedies, we will find that there are two kinds of treatment, strongly distinguished from each other, applicable to certain classes of cases respectively. General hygienic treatment is applicable to all; and this will consist, for the most part, in an abstinence from every thing that stimulates the ovaria, heated rooms, high living, lascivious practices, marriage, or if married, sexual intercourse and pregnancy; and the use of appropriate out-door exercise, proper diet, and, indeed, everything calculated to promote sound health, and an avoidance of all debilitating influences.

The two kinds of treatment alluded to are the *palliative* and the *curative.* The one intended to relieve, as far as possible, the sufferings of the patient under the disease, or to retard the rapidity of its progress; the other, to remove, or destroy the tumor, and thus do away with the cause of the evil entirely. When practicable, I think this last treatment is always desirable; when not practicable, as, unfortunately, is too frequently the case, our only resource is the former.

So far as my influence goes to enforce the rule, I should emphatically insist upon determining in the beginning, before we make a move toward treatment, the kind of means adapted to the case under consideration; institute that treatment, and persevere with it to the end, or until some circumstance occurs to convince us of our mistake in this respect. We should not begin by trying what is usually regarded as the least hazardous, and be ready to use the more dangerous, but yet more effective, *because the former had failed.* There is no treatment worth relying upon, but must produce its effects; and in nine cases out of ten, if it does not cure, it will cause disagreeable, and even dangerous complications, and so render the subsequent treatment less effective. No case in which tapping, injections, or efficient pressure has been used ineffectually, is ever left in as good a condition for extirpation, as before any such interference. Hence, I would not tap a tumor that ought to be extirpated, nor allow it to be injected or compressed. It may be said, and it is unquestionably true, that we may not always be able to determine the character and complications of the tumor, so as to be certain which treatment is best adapted to it. In answer to which, I would say, we must do the very best we can in this respect; and I think by thus forming and acting upon our judgment, more lives will be saved than by confounding the remedies, in the manner heretofore done, using them more with reference to their danger and efficiency, than their adaptation to the particular case. It would seem that practitioners are divisible into two classes, as to their mode of treating ovarian tumor, viz: those who believe that radical treatment should almost always be avoided, and those who believe in operating in nearly every instance.

Two prolific sources of embarrassment in the treatment of ovarian diseases are first, the knowledge, on our part, of their almost invariably fatal tendency; and second, the inability of our patient to appreciate the appalling nature of her condition, until such constitutional effects have been produced as would render any efficient treatment powerless to save,—at least, this has been the case with my patients to some extent, and hence we should make a clear and decided statement to them so soon as we become able by our investigations to do so. When doubt exists as to the propriety of instituting radical treatment, we should continue to pursue the palliative until that doubt is dispelled. There are three sorts of cases to which the palliative is indisputably adapted. They are first:—those in which it is not desirable to use radical means in consequence of the absence, and probable great distance of urgent symptoms, while there is a steady advance. It may as well be remarked here, that no palliative measure, such as tapping, great pressure, or other means that will render radical treatment more hazardous should be employed, where we are likely in the future to desire to resort to operative procedure. The second class of cases is that in which the symptoms are urgent, but in which it is not desirable to use radical means, in consequence of the slight chances of success. The third set are such as, in their nature and condition, would call for curative means, but the patient will not consent to their employment from fear of the danger or pain they inflict. The first set of cases is not very frequently met with, compared to either of the others; yet we do occasionally meet with these slowly marching cases, in which we have an opportunity to try the effect of medicines; and it is precisely in this kind of cases that we *appear* to derive most benefit from medicines internally administered. We are prone to believe that the tardy development is dependent upon the virtue of some favorite remedy used, and deceive ourselves as to its efficiency; when really all depends on the natural slowness of the tumor. The alteratives, as mercury, iodine, sarsaparilla, chlorine, etc., have all had their advocates. It was at one time, and even now is the practice of some men of ability, to give mercury to very slight ptyalism, with the hope of bringing about absorption. Iodine administered frequently, so as to induce its specific influence upon the organism, has been, and is still, by some, highly lauded as capable of curing ovarian dropsy. A chronic administration of either of these remedies is sure to affect unfavorably the general health; and as it is extremely doubtful whether there is any efficacy in them, we should not be too profuse in their use. Effusion into the peritoneal sac, or sub-acute inflammatory complications, are often very much benefitted by a moderately protracted course of these remedies. For the same purpose, local depletion, counter-irritants, such as iodine ointment, strong enough to induce irritation of the skin, are often useful; so are diaphoretics, diuretics and cathartics. But, probably, the best course to pursue, in these cases, is to keep up the general health by appropriate exercise, diet and tonics when necessary; and to keep the abdomen constantly, but gently compressed by a flannel roller, which should be applied all over the abdomen, but applied with more stringency over the tumor, and to add to its efficiency, a compress of sufficient thickness may also be employed. I must be permitted again to say, with reference to all these cases, that until the necessity for active interference, in consequence of the urgent effects of the growing tumor is fairly presented, we should carefully avoid all very strong pressure, or operative procedure. In the second class of cases, we need not feel so restricted in our efforts at palliation; it is best, however, to bear in mind, that too great activity of medication will often do more harm than good. Our object should be to promote such functions as are obstructed, or restricted; the kidneys, for instance, will need especial attention, as will the intestinal canal. The acids have always seemed to me to be particularly applicable to these cases; the nitric, nitro-muriatic, sulphuric, phosphoric, acetic, are all good, and may be alternated often, with the hope of relieving the distressing indigestion attendant upon great distension and imperfect performance of the renal functions. They also very much moderate the distressing exudations from the skin which are often present. The chlorinated tincture of iron is an excellent tonic in such cases. These remedies may all very properly be administered in some of the bitter infusions, quassia, camomile, wild-cherry bark, etc. The best time to give them is immediately after eating. Stimulants ought not to be too freely used, as they encourage the establishment of complications. Brandy, I think the best of the stimulants, and it should be given more for the purpose of inducing sleep, than anything else; and this it will often do, when taken in a sufficient dose on an empty stomach, at bed time. When great restlessness, and want of sleep are wearing out the patient, we must, as in all similar circumstances in other diseases, resort to the assortment of anodynes, beginning with the less disturbing,—being sure to be under the necessity of ending with opium. Chloroform, internally administered, is, I am confident, not sufficiently relied upon. Teaspoonful doses, given in milk, will seldom fail to induce a fine anodyne effect. There is greater necessity perhaps, for a gradual increase of the dose in using it than opium, or most other efficient anodynes. Hyoscyamus, belladonna, cicuta, should all be tried before opium.

We must be on the alert for other complications, and ready for their appropriate treatment. The distressing constipation, which often annoys the patient and physician, will demand a great share of our attention. Injections of water, and various substances will, of course, suggest themselves. It has occurred to me, to be able to induce free movements of the bowels, by having a pint of warm lard thrown high up in the bowels, when they are very obstinate; the longer the lard is retained, the better. This administered once a day, will act excellently well sometimes. An ounce of fresh beef's gall with three or four ounces of water, often does as well. But the time comes, sooner or later, with the steadily increasing pressure of the tumor, when to lessen its size is indispensable to the further extension of life. Tapping suggests itself as the only palliative means in this state of things. This operation is more beneficial in unilocular tumors than in any other sort; but, is applicable, as a palliative measure, in any tumor containing fluid, when demanded by the supervention of urgent symptoms, indicating the necessity of immediate relief. Under the desperate circumstances mentioned, there can be no question about the propriety of tapping the patient, yet this apparently trifling operation is not devoid of inconveniences and dangers, that should be weighed deliberately, and if they do not deter us from resorting to it, will at least make us particular not to use it as anything but an indispensable remedy. One serious inconvenience connected with tapping, is the readiness with which the fluid re-accumulates in the sac; indeed, the oftener the patient is tapped, the more rapidly will accumulation take place. If, therefore, we commence using it too early, we are likely to shorten the life of the patient, if in no other way than by the increasing emaciation, dependent upon the immense drain of fluid from the system.

The dangers of tapping are both immediate and remote. The immediate are such as are connected with, and occur immediately upon the performance of the operation. Dr. Simpson sums up five that are more frequent, and against which we should be upon our guard;—first, the chance of wounding the urinary bladder: this may be avoided by evacuating the organ, unless it is tied to the abdominal wall by adhesions; second, the puncture of the uterus when it is drawn up with the tumor: by introducing the sound into its cavity we may learn its whereabouts, and thus be enabled to avoid it; third, the front part of the tumor may be traversed by the fallopian tube, and this last be wounded by the trochar,—an accident which should, of course, be avoided when we can, but which I cannot think of much importance, nor do I know how it can certainly be avoided; fourth, the internal venous circulation, on account of the pressure, is obstructed sometimes, and the blood is directed to the veins in the walls of the abdomen, so that these veins may be wounded, but generally they are large, and may be seen, and thus avoided; fifth, the epigastric artery is sometimes wounded: we should carefully feel for the pulsation of arteries in the thin walls before the trochar is plunged into the tumor. As may be seen, these dangers, may, for the most part be provided against; but the second class of dangers,—namely, the remote,—those that follow the operation some time after its performance, and are not dependent on the manner or place of the puncture, are unavoidable, and cannot be foreseen.

The dangers and benefits of tapping cannot, and ought not, to be estimated by comparison with other operations. Each operation of whatever kind, has its place, and is followed by its good or bad effects, for the reason, among others, that it is appropriate, or inappropriate. Generally, no two operations are applicable to any one condition of things; and we should not allow the question of danger to decide between them, unless in very rare and exceptional cases. The statistics, as far as I have been able to collect them, may be well summed up, as Dr. West has done, and I shall rely upon his figures: "The chief, indeed, almost the only, numerical data of which we are possessed, bearing on this subject, are derived from a table of 20 cases, compiled by Mr. Southam, of 46 cases collected by the late Mr. Lee, and of 64, the results of which are given by Professor Kiwisch. Of these 130 cases, 22 terminated fatally within a few hours or days after tapping, and 25 more in the following six months; or in other words, 34.7 per cent of the cases ended in the patient's death in the course of half a year after the performance of tapping. In 114, of the 130, death is stated to have taken place: 22 within less than ten days, 25 within six months, 22 within one year, 21 within two years,

11 within three years, 13 after a period exceeding three, and in some amounting to several years.

In 109 of these cases, we are further informed how often the patients had been tapped. It appears that 46 died after the first tapping, 10 after the second, 25 after from three to six tappings, 15 after seven to twelve, 13 after more than twelve." It would appear that the first tapping is very much more dangerous than subsequent ones. Dr. WEST says further:—"unfavorable, however, as are the conclusions to which we are irresistibly led by such facts, such as those which have just been mentioned with reference to the ultimate issue of tapping, it is yet very questionable whether they represent the whole of the truth concerning this matter." Dr. ATLEE, of Philadelphia, thinks tapping not a very dangerous operation. Mr. BROWN thinks its dangers greatly overrated.

There can be but little doubt that much of the mortality of tapping is due to the fact of the desperate character of the cases in which it is used; and the reason why so many die in so short a time after the first operation, is, that in many instances the patient is almost moribund before it is resorted to. When not attended with the immediate dangers above enumerated, tapping is either followed by great relief from suffering or by the remote or sequential dangers. They are for the most, prostration or inflammation. The prostration is sometimes so great, that no management can prevent the patient from dying in a very short time. Such great prostration is, however, exceedingly rare; it is more common to have it in a more moderate degree. The patient will feel faint for an hour or two, and then gradually rally, or she may continue to be pale and languid for several days. For such slight cases, the horizontal position, rest, and good, digestible, somewhat stimulating food is all that will be needed. When the prostration is great, and danger of fatal sinking present, the case must be treated energetically. The means calculated to bring about reaction must have reference to the causes of the prostration. The evacuation from the general vascular system is not a cause, because the fluid in the tumor is extravascular; but it is a sudden change in the distribution of the blood. The evacuation of the abdominal cavity of so large a bulk of its contents, and the inability of the abdominal muscles to contract sufficiently to keep up the pressure to which the viscera have been habituated, are the causes of the irregular distribution of the blood. The want of pressure upon the abdominal viscera allows a large accumulation of blood in their veins, and it is there retained. In proportion to the amount thus collected in the abdomen, will the blood be withdrawn from other parts and organs. The brain will partake of this temporary anemia, and consequently be incapable of discharging its functions with its wonted efficiency. This is the condition, not a want, but an irregular distribution of blood. Our first object should be to, as nearly as possible, rëestablish the previous condition of the abdomen. This can be, to some extent, accomplished by pressure, with compresses and rollers. The compresses should be as large as the space covered by the muscles of the abdomen, and thick enough to fill up much above the level of the ribs and iliac bones on the side. The roller should be applied from the pubis to the ensiform cartilage, with as much power as the patient can bear, without great discomfort. Then the head should be persistently kept below the level of the body. This simple treatment, instituted early, will do more than all other means without it. We can very properly, however, give stimulants, in addition, when necessary. When this danger is passed, inflammation of the sac or peritoneal cavity is next to be apprehended. The sac undergoes every degree of inflammation, from the slow, subacute, unobserved degree, which vitiates the fluid effused into it, either by allowing decomposition in it, or by the production of pus, or effusion of blood inside, or fibrin on its external surface—in this last case causing adhesion—or such degeneration of the walls of the sac, as to cause an obliteration of the cavity, a cession of its secreting powers, or a perforation, and consequent peritoneal communication; or, what is perhaps more common, an acute degree, announced by severe pain referred to the point most intensely affected, or to the whole abdominal region, thus showing the probable involvement of the peritoneum. Indeed, I think it very probable that the sharp pain ordinarily present in these cases, indicates peritoneal inflammation, and that there is but little pain in the case of inflammation of the fibrous and internal coats of the sac. Fever, of a somewhat high grade, is apt to attend upon the degree of inflammation last mentioned, accompanied by headache, weariness, aching in the back, limbs, etc. But in the inflammation of the inner coats, in which pus or fibrinous products are effused in the fluid of the tumor, there is generally but slight fever, perhaps none, at first; but the vital powers are more or less depressed, copious perspirations at night, possibly delirium, and in bad cases, all the symptoms of pyæmia, hectic, exhaustion, and death. Now all morbid conditions resulting from tapping, should be met promptly by the remedies appropriate to them, when they occur under other circumstances,—antiphlogistic regimen, depletion, fomentations, cathartics, anodynes, alteratives, etc. In pyæmia, tonics, stimulants, good diet, and time will be our resort.

The operation of tapping is simple, and easily performed generally. To avoid the depression which follows the evacuation of so large a quantity of fluid as is contained in the abdomen sometimes, we should have our patient on the side, very near the edge of the bed, with her head and shoulders low. Two large and long hand towels should be passed around her body, with the edges close together upon a level with the point where we wish to introduce the trochar, and these ends given to an assistant who stands behind the patient. The assistant, having in charge these hand towels, should be directed to draw upon them so as to keep up a state of tension as the fluid is being evacuated. To avoid the dangers enumerated as immediate, we should assure ourselves that the bladder is empty, and if we mistrust that it is not in its proper place, we should introduce a sound, so as to assure ourselves of the whereabouts of the fundus. If we have not already done so, we *must* sound the uterus, also, and thus be sure of its harmless position. After these precautions, the best rule, perhaps, is that given by Dr. SIMPSON; and that is, to feel for the most fluctuating point, the place where the walls are thinnest,—look for veins and feel for the pulsation of arteries. The thinnest parts, where fluctuation is most evident, is usually the right place to make the puncture; but there is not always any such point, there being but little difference in this respect over the whole of the front surface of the tumor. In such case we may be governed by the ordinary rules for the place for tapping. The linea alba, between the symphisis and umbilicus, is the most eligible in the greatest number of cases. If any objection to this arises, a point midway between the umbilicus and the anterior superior spine of the ilium is, as a general thing, safe and effectual as any. Some surgeons recommend other places as free from the objections that are sometimes urged against these points. They say that tapping through the vagina is quite safe from the immediate, and not so likely to be followed by some of the sequential disasters. The rectum is thought to be still better by some. The vagina is quite a commendable place, if we are careful to ascertain well the position of the bladder and uterus, and avoid them. Our instrument (the trochar) should be large, four or five lines in diameter, the point should be sharp, and a little longer than they are usually made. The canula if not large, will not freely discharge the fibrinous concretions, or thick treacle-like fluid, and if the point is not long and sharp, we inflict considerable unnecessary suffering in the introduction of the instrument. We may plunge the instrument in towards the central axis of the tumor, until sent home to the rim of the canula. If, however, our instrument is not pretty sharp, it will be very much better to make an opening with a very sharp, thin bistury, which will cause less suffering, and answer every purpose as well.

The third sort of cases to which palliative treatment is applicable, those in which our patient will not submit to radical means, must be managed in almost every particular as I have described the treatment for the other two kinds. Remembering the rules and rationale, it will not be difficult to adapt our means to the end in view.

The curative treatment of ovarian disease is believed by many, almost all, authorities, to be practicable only by instrumental means. There are some very respectable writers, however, who believe that there are cases in which we may hope for success from medicinal and mechanical treatment without the use of surgical instruments, and they think that there is enough virtue in such means to warrant a trial in very many instances.

The immediate objects to be accomplished are, first, to arrest the growth of the tumor; bring it to a stand-still; and thus avoid the disastrous results which attend the attainment of very large size, with its consequent pressure, ruptures, etc. Second, when this is not practicable, to obliterate the sac, or otherwise the tumor. The sac is sometimes reduced by contraction to a mere knot of compressed tissues, which are more and more atrophied and wasted, until very slight traces of their existence are left; or, by inflammation and contraction, the tumor is converted into a fibrinous mass, enveloped in a fibrinous sac, which remains the same throughout life, with very little alteration; or suppuration may accompany inflammation,—the whole tumor be softened down into pus, and discharged by ulcerating through the vagina, rectum, abdominal walls or bladder, and all traces of it disappear. Or, again, the walls may collapse without shrinking much, and adhere by adhesive inflammation, and in this way its effusive surface be destroyed.

When neither of the above two immediate objects is practicable, or it may not be desirable or advisable to attempt them, we may (thirdly), remove the whole, or a part of the tumor from the abdomen, and thus either get rid of the whole of the offending growth, or, after a part is removed, hope to effect, by one of the processes of obliteration above alluded to, the destruction of the balance. The means used for the arrest sometimes cause an obliteration of the sac, and do more than merely stop its growth; so that it will not be the best plan to formally treat of those means by which we attain the first object desired. I shall consequently feel at liberty to introduce and speak of such as sometimes arrest, and sometimes cause a disappearance of the tumor.

Three general ideas seem to govern individuals who rely largely upon the use of internal remedies for the cure of ovarian dropsy, viz: that the disease is inflammatory in its origin and continuance, and that antiphlogistic and alterative remedies, by arresting this morbid process, will stop its growth, and that the conservative powers of the system, aided by sorbefacients and secernents, will remove it:—that the tumor is developed in consequence of the presence of some one of the cachexiæ, scrofula, for instance, as Dr. BIRD distinctly avers; physicians who entertain this notion of its origin hope to make a cure by changing the general action of the system, by all the means usually recommended for the correction of scrofulous tendencies to disease,—by tonics, good diet, properly regulated exercise, clothing, bathing, and specific medication:—that its origin is entirely independent of either inflammation or scrofula,—a hypertrophy, in the strict sense of the term, of certain normal conditions,—a nutritional development of tissues, similar to the production of nutrition elsewhere; those who entertain this doctrine believe, also, in the atrophicating qualities of certain medicines and mechanical appliances, and hope, by the well directed employment of them, to at least arrest their growth, if not cause their removal by absorption. A number of cases are on record that encourage the hope of doing something by medicines internally administered; and while I am free to state that I have really hardly any faith in them, as curative means, the recollection of the discouraging results of any kind of treatment, forbids me too strongly deciding against their use in properly selected cases. Nor do I think it fair to say, as has been said, that cases treated by internal medication and recovering, are but instances of spontaneous cures, and would have done as well or better without the treatment. Dr. DENMAN, in his midwifery, by Dr. FRANCIS, at page 151 says, "in the beginning of this dropsy, when the increasing ovarium is first perceptible through the integuments of the abdomen, and sometimes in its progress, there is often so much pain as to require repeated local bleeding by scarification or leeches, blisters, fomentations, laxative medicines and opiates to appease it. I have also endeavored to prevent or remove the first enlargement by a course of medicines, the principal of which was the unguentum hydrargyri rubbed upon the part, or calomel given for a considerable time in small quantities with an infusion of burnt sponge; or the ferrum tartarisatum or ammoniacal; trying occasionally what advantage was to be obtained from blisters, from a plaster of gum ammoniacum, dissolved in the acetum scillæ, or lastly, from electricity. From all, or some of these means, I have frequently had occasion to believe some present advantage was obtained, or mischief prevented; but when the disease has made a certain progress, though a variety of medicines and of local applications have been tried, no method of treatment has been discovered sufficiently efficacious to remove it, or prevent its increase."

Colombat is of the opinion that "though we ought not to place much confidence in the means derived from medicine, strictly so called, we are of opinion that they ought always to be employed before recurring to those offered by surgery. Consequently sudorifics ought first to be prescribed, for example: guaiac, sarsaparilla and vapor baths; resolvents, and amongst them mercurial frictions successfully employed by Clark and M. Nauche; hydiodrate of potash, with the internal use of iodine in small doses; sea-bathing, or salt water baths, from which M. Laennec, of Nantes, says he has obtained most excellent effects; the thermal baths of Aix, in Savoy, or those at Barege, and lastly, antimonial frictions, cauteries, moxas, and blisters, applied upon the abdomen. Diuretics, such as squills, nitre, etc., which, according to Haller were usefully employed by Willis; a decoction of ashes, in the proportion of a handful to the quart of water, employed by Petit Radel, and from which he obtained a cure after having punctured the cyst. Lastly, purgatives in divided doses; as for instance, aloes, rhubarb, croton oil, calomel combined with castile soap and sulphate of potash, etc., are other means, which, in conjunction with abstinence and compression of the abdomen, may be prescribed at the commencement of the disease for the purpose of assisting the absorption of the fluids, at first small in quantity." After trying all these, however, surgical treatment, he thinks, will be our only resort in a vast majority of cases. The efficient application of pressure seems to promise more than internal remedies. Well regulated, efficient and long-continued pressure may produce obliterating inflammation of the sacs, and consequent cure; or, by affording great resistance to the expansion of the tumor, arrest its growth.

Resolution and absorption of an ovarian tumor is a very doubtful fact however, and notwithstanding their unaccountable disappearance, should not be counted prognostically. The second object in our treatment, that of obliterating the sac *in situ*, affords more reason for hope in properly selected cases. The means used consist of tapping, with pressure, with injections of stimulants to induce inflammation of the sac, and with injections and pressure combined; or what is sometimes successful, the establishment of a fistulous opening in the sac, that either communicates externally, through the abdominal walls, through the vagina or rectum, or simply with the peritoneal cavity. The above mentioned treatment is applicable, properly, to the unilocular or single cyst cases only, as it is impracticable to tap, inject or establish a fistula when there are many sacs; and what is still more discouraging in the multilocular variety, the sacs are not only filled again after tapping, as is generally the case with the monocyst; but there is a constant reproduction, or perhaps it would be more correct to say, that they are continuously developed from the ovisacs that are matured every month. Tapping, followed by pressure or injection, is very apt to change the condition of the tumor, in one respect at least, and that is to cause adhesions to the surrounding peritoneal surface. In one case of unilocular tumor, in which an external fistulous opening was made after the patient had been tapped six times, and had iodine injections three times, the sac, so far as we could determine, was universally adherent,—no portion of it could be brought out of the wound.

Very fortunate instances sometimes occur, in which the evacuation of the tumor by tapping is followed by a speedy and permanent obliteration of the sac. It is exceedingly doubtful, however, whether these were not cysts developed from the broad ligament, or some substance in the neighborhood of the ovary, but not involving its tissues at all. Certainly, they are exceptional, and cannot be expected in any given case; so that we ought never to be satisfied with tapping, when our object is the obliteration of the cyst. Pressure, in conjunction with tapping, is applicable, perhaps, to a larger number of cases than any of the other modes of treatment. It is very much more successful in cases of the monocystic than in any other variety. The application of pressure to a tapped sac, has for its object a complete closure of the cavity of the cyst in such a manner, as to bring its walls as nearly as practicable, in contact throughout; this at once, if thoroughly effected, modifies the secerning capacity of its surface, and perhaps from the time of its application, arrests, more or less completely, the effusion of the fluid. Now, if this cannot be done so as to operate upon all the surface of the walls, we can almost always bring some portion of the collapsed walls in contact. The continuous and prolonged contact of these surfaces bring about a low, and in some cases a pretty high grade of inflammation, causing adhesion, or a change in their structure, so that they are no longer of the same ovisac nature, and hence they do not effuse the thick albumen previously produced, and the tumor remains inactive, or shrinks and nearly, or entirely disappears; or suppurative inflammation may dissolve down, and discharge the mass through some adventitious or natural outlet.

(*To be Continued.*)

